# Range-compensated pencil beam scanning proton Arc therapy: a feasibility study

**DOI:** 10.1038/s44172-025-00460-z

**Published:** 2025-07-31

**Authors:** Blake R. Smith, Ryan T. Flynn, Alonso N. Gutiérrez, Daniel E. Hyer

**Affiliations:** 1 Department of Radiation Oncology, University of Iowa Health Care, Iowa City, IA 52242 USA; 2 Department of Radiation Oncology, Miami Cancer Institute, Baptist Health South Florida, Miami, FL 33176 USA

**Keywords:** Radiotherapy, Biomedical engineering, Radiotherapy

## Abstract

Proton arc therapy is a conceptual treatment technique in proton therapy that delivers a scanned proton pencil beam simultaneously as the gantry is rotated around the patient, exploiting the geometric advantages of a continuous arc with the dosimetric advantages of protons to maximize healthy tissue sparing. Here we propose an alternative approach to deliver pencil beam scanning (PBS) proton arc therapy using a beam-modifying device called a SpeleoFilter. SpeleoFilters can improve the treatment efficiency of proton arc by reducing the number of beam energies and beam spots while preserving the plan quality as compared to traditional multifield intensity modulated proton therapy (IMPT). The proposed SpeleoFilter framework was validated within a state-of-the art PBS collimator and IBA Dedicated Nozzle PBS system at the Miami Cancer Institute. The Monte Carlo methods developed in this work showed great agreement with experimental measurements and matched depth dose profiles within a 1–2%/1 mm gamma criteria. Proton arc treatments utilizing a SpeoleFilter resulted in comparable healthy tissue sparing and an order-of-magnitude reduction in the number of energy layers compared to IMPT for both phantom and patient datasets. Further work is necessary to fully demonstrate its application and clinical integration for multiple treatment sites.

## Abstract (web summary)

Blake Smith and colleagues propose an innovative approach to pencil beam scanning proton arc radiotherapy using customized range compensators. These devices were optimized to omit energy changes while improving treatment deliverability and efficiency.

## Introduction

Combining pencil beam scanning (PBS) with an arc-style delivery, referred to as proton arc therapy (PAT), is an experimental treatment technique that combines the dosimetric advantage of proton therapy with the geometric advantages of an arc delivery^1^. Recent studies investigating proton arc to that of fixed-field intensity modulated proton therapy (IMPT) have demonstrated synergy of utilizing an arc-style delivery with PBS to provide a more robust delivery that mitigates beam range and stopping power uncertainties on the plan quality^2–4^, has the potential to reduce the total treatment time^5^, and is further enhanced when combined with energy specific collimation^6^. However, the physical delivery and clinical implementation of PAT remains conceptual as the distribution and sequencing of energy layers is difficult to optimize as only a single energy may be delivered at a time. Simply increasing the number of beam energies may compromise the treatment delivery time due to the increased number of energy changes and be limited by the minimum deliverable monitor units for each beamlet. Even with the advent of sophisticated energy switching algorithms, the total delivery time can be prolonged due to the numerous energy changes required to cover the target across a wide angular span^3,7^.

Historically, passive scattering technologies expediently delivered multiple beam energies in proton therapy using broad beam modulators and reshaped downstream to the radiological projection of the target using a collimator and compensator^8^. Such techniques, like the modulation propeller wheel and ridge filter, are more difficult to adapt with active scanning proton delivery systems as beam modulation would occur upstream of the scanning magnets. Recent developments in modulator design have led to the development of mini ridge filters for some PBS and proton FLASH applications^9–12^, which are limited to spatially invariant dose profiles. As such, their use in PBS is employed to broaden individual pristine Bragg peaks or create spread-out Bragg peaks (SOBP) for low-energy beams or to achieve a desired dose rate and dose distribution for FLASH applications^13^. Some recent works have proposed the use of a 3D range modulator to deliver 3D conformal treatments^4,14^. In principle, the design of the modulator is specific to a particular treatment field, utilizing an array of telescoped tubulars to deliver a spectrum of proton energies from a single mono-energetic scanned proton beam, which is confined to only a statically delivered treatment field.

The scope of this work aims to expand on the existing literature of external modulation devices in PBS by developing a new approach to delivering PBS PAT. Inspired from historical and current technologies, a beam filtration device, referred to as a SpeleoFilter, is proposed to enable efficient delivery of PAT and other applications of PBS that require the expedient delivery of multiple energy layers while preserving a high degree of plan quality and delivery flexibility associated with PBS and proton arc therapy. Conceptually, the device can be thought of as a heterogenous block of material consisting of pillars of cross-sectional dimensions much smaller than the lateral extent of a proton beamlet that vary in height protruding from the base. The distribution of pillars is optimized across the entire SpeleoFilter to produce a spatially variant, polyenergetic proton spectra resulting in the desired dose distribution for the treatment with as little as a single beam energy. Such a technology would extend the current clinical practice, be compatible with other synergistic technologies while also generalizable among different vendors or delivery systems. This study presents (1) a method to optimize the design of a SpeleoFilter for various applications, (2) experimental validation of the Monte Carlo models used to optimize a SpeleoFilter, and (3) a feasibility study demonstrating proof-of-concept optimization of SpeleoFilters to deliver PAT.

To demonstrate feasibility, an initial set of SpeleoFilters were designed to provide specified SOBP widths, which were experimentally verified to benchmark the computational model. As shown in this work, the compact design of these filters enabled their integration with a state-of-the-art PBS collimator, the DCS, using a custom mounting prototype secured above the collimating blades, which could be used to improve the target conformity and healthy tissue sparring. The treatment planning studies performed in this work demonstrate the feasibility of these devices to be optimized to achieve a desired proton dose distributions for both a phantom and a patient dataset. While the results from this work are promising, there remains a notable amount of work necessary to fully demonstrate and integrate this approach to proton arc.

## Methods and materials

### Monte Carlo modeling

Monte Carlo simulations were performed using the Monte Carlo toolkit Geant4 (Version 4.1005)^15^. Simulation executables were compiled from native or customized class structures in C + + that were called by, and run in parallel with, the MATLAB SpeleoFilter optimization. The charged particle energy loss for all simulations was determined using the standard g4h-phy_QGSP_BIC_HP hydronic and g4em-standard opt4 electromagnetic transport physics package. Nuclear interaction and uncharged radiation transport physics were registered to the run manager throughout the G4VModularPhysicsList class using the standard QGSP_BIC_HP package available in Geant4. Absorbed dose to medium was tallied in a voxel array using the G4Primitive Scorer to sum energy depositions and record the statistical variance.

A custom divergent point source was modeled after the Ion Beam Applications (IBA) Dedicated Nozzle (DN) Proteus One beam line at the Miami Cancer Institute (MCI) in Miami, Florida. Spot divergence was modeled following the Monte Carlo techniques of Smith et al.^16^, Gelover et al.^17^, and Nelson et al.^18^. Beam line-specific parameters for the IBA DN system and modeling of the beam detection while scanned using an X- and Y-bending magnet were adopted from experimentally benchmarked dynamically collimated Monte Carlo (DCMC) package of Nelson et al.^18^., which accurately models the double-focused alignment of the trimmers in the Dynamic Collimation System (DCS) to each scanning magnet^19^. Using these methods, the beamline optics are mathematically accounted for in the source definition, resulting in excellent spot profile and PDD agreement on and of axis. It should be noted that these Monte Carlo methods are built upon an existing simulation architecture that has been used extensively for system prototyping of the DCS^19^ and in the development of analytical calculation algorithms to be used for DCS treatment planning in an FDA-cleared treatment planning system^20^. The use of this modeling framework also enables a compatible framework with the DCS, a state-of-the-art PBS collimator.

SpeleoFilters were modeled as a heterogenous block of material of pillars with fixed cross-sections and varying heights protruding from a common base layer. Protons traversing through the SpeleoFilter will encounter differing amounts of the SpeleoFilter material between adjacent pillars that is dependent on their trajectory through the SpeleoFilter. Given the proprietary nature of VeroClear (Stratasys, Eden Prairie, MN), a nominal elemental composition of adipose tissue defined in ICRP 110 was assumed based on the x-ray spectroscopy results from Santos et al.^21^. and included a relative chemical composition by weight of 11.4 %, 58.9 %, 0.7%, 28.7%, 0.1%, 0.1% and 0.1% for hydrogen, carbon, nitrogen, oxygen, sodium, sulfur, and chlorine, respectively. An initial density of 1.19 g cm^-3^ was assumed based on the manufacturer material specification report for VeroClear provided by the manufacturer. Dosimetric studies were performed for two phantom geometries in this work including a simplified water phantom and a patient dataset. Simulated treatment deliveries were calculated using the patient dataset by reconstructing the voxelized geometry of the patient within the modeled beamline framework that included the DCS and Speleofilter system. Sensitive detector volumes that tallied dose deposition events were instantiated for each patient voxel, retaining the equivalent spatial resolution as the voxelized patient geometry. Tissues modeling parameters were defined using a CT Hounsdfield unit to material definition model from Schneider et al.^22^. Simulations were run with enough histories to within 1% statistical uncertainty.

### Experimental validation

**Fig. 1:**
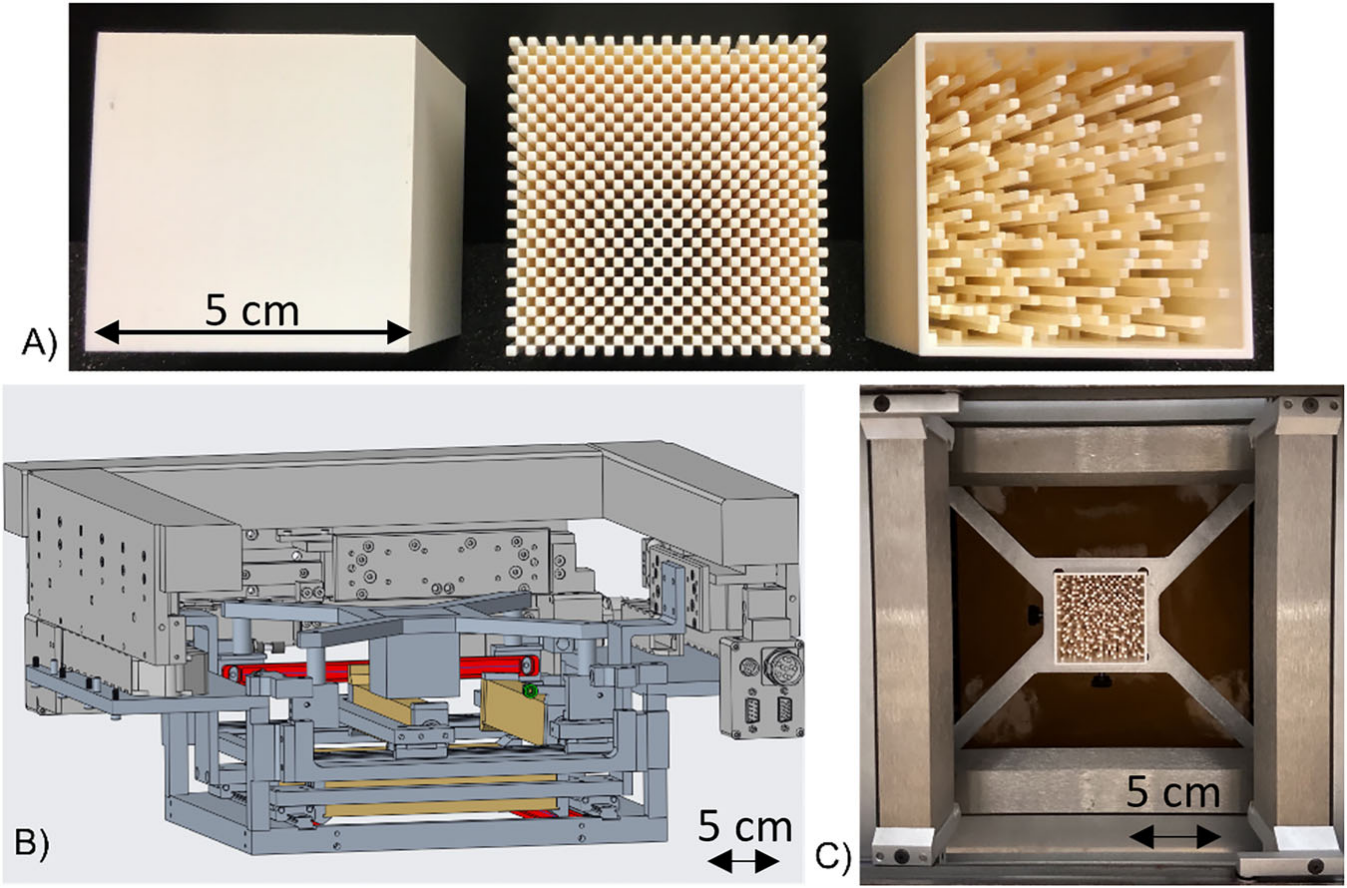
Experimental prototype SpeleoFilters and their integration within a preclinical PBS dynamic collimation system. **A** The experimental and prototype SpeleoFilters studied in this work: solid block (left, SpeleoSolid), pre-planned pillar distribution of alternating 50 mm and 3 mm pillars (center, SpeleoPillar), and an optimized design providing a uniform dose profile (right, SpeleoOptimized). **B** CAD model rendering of the dynamic collimation system with the accessory amount for the custom SpeleoFilters. **C** Photograph of the SpeleoFilter inserted upstream of the collimating trimmers inside the DCS mounted within the IBA DN system at the Miami Cancer Institute.

Dosimetric modeling of the SpeleoFilter system was experimentally verified at the Miami Cancer Institute’s (MCI) IBA (Ion Beam Applications, Louvain-la-Neuve, Belgium) Proteus Plus Dedicated Nozzle (DN) PBS system. A set of three prototype SpeleoFilters were 3D printed using a customized polyjet 3D printing technique from Stratasys (Eden Prairie, MN) that allowed for high-precision 3D-printing with a tolerance of 0.127 mm without the use of supportive wax. SpeleoFilters were mounted within a custom holder that was secured to the DCS in place of the external range shifter, upstream of the collimating components shown in Fig. 1. Each filter served a specific purpose to (1) benchmark the radiological properties using a solid block (SpeleoFilter referred to as SpeleoSolid), benchmark Monte Carlo transport methods and experimental sensitivity (SpeleoPillar), and experimentally demonstrate, via proof-of-principle, the planning and integration of an optimized SpeleoFilter (SpeleoOptimized). As the SpeleoSolid filter consisted of a uniform, solid block, differences in material density would have dominated any discrepancies between the Monte Carlo simulated profile and what was measured while being insensitive to small alignment offsets. The SpeleoPillar filter was designed with a repeating pattern of 50 mm and 3 mm pillars to further refine material definition as well as study the alignment sensitivity of the SpeleoFilters to the incident proton beam. By design, the resultant depth dose profiles from the discretized pillar heights are much more susceptible to alignment errors, which alters the relative intensities of the two observed Bragg peaks. These features were used in the analysis of the profiles to discriminate between setup errors and nominal material composition differences between the simulated and measurement conditions. The spatial characteristics of the SpeleoOptimized filter were optimized to produce a 5 cm SOBP using the mathematical methods developed in this work.

An initial quality control check of the manufacturing accuracy and density of the SpeleoFilters was performed using a high-resolution, 0.6 mm slice thickness, CT scan of each SpeleoFilter with a 0.5 mm lateral resolution acquired on a Siemen’s Biograph PET-CT scanner. The surface of the SpeleoFilter was auto contoured in Pinnacle by auto thresholding (Version 16.2) and was exported as a DICOM structure set. A custom MATLAB application was created to overlay the CT-drawn surface contour against the original optimized SpeleoFilter design and quantify the manufacturing accuracy through computing the DICE similarity coefficient between the two surface contours and reporting the mean Hausdorff distance. The density of the VeroClear material was measured from a central region of interest within the solid SpeleoFilter block.

Simulations and measurements were performed with the DCS mounted to the telescoping nozzle to produce a 5 cm $\times$ 5 cm collimated field, defined as the projected field size at isocenter to block low-energy spray scattering out from the SpeleoFilter. Measurements were performed using a 150 MeV $5\,{\mbox{cm}}\,\times \,5\,{\mbox{cm}}$ uniform field, which was initially benchmarked for a pristine proton beam delivered using a 2.5 mm spot spacing. For each SpeleoFilter, central axis percent depth dose (PDD) distributions were measured in water using an IBA PPC05 plane parallel ionization chamber that was secured to the translational stage of an IBA DigiPhant using an in-house chamber mount. Given that the depth dose characteristics of the SpeleoFilter were optimized from a uniform field, PDD measurements were acquired by delivering the entire field at each measurement depth. Consequently, PDD measurement points were prioritized around prominent features of interest and were normalized to the reference depth of 4 cm within the plateau region. The measured PDD and lateral profiles were then compared against Monte Carlo calculations that modeled the placement of the SpeleoFilter within the DCS and MCI beamline using a gamma analysis test with varying gamma criteria.

### SpeleoFilter design and optimization studies

#### Phantom planning study

**Fig. 2:**
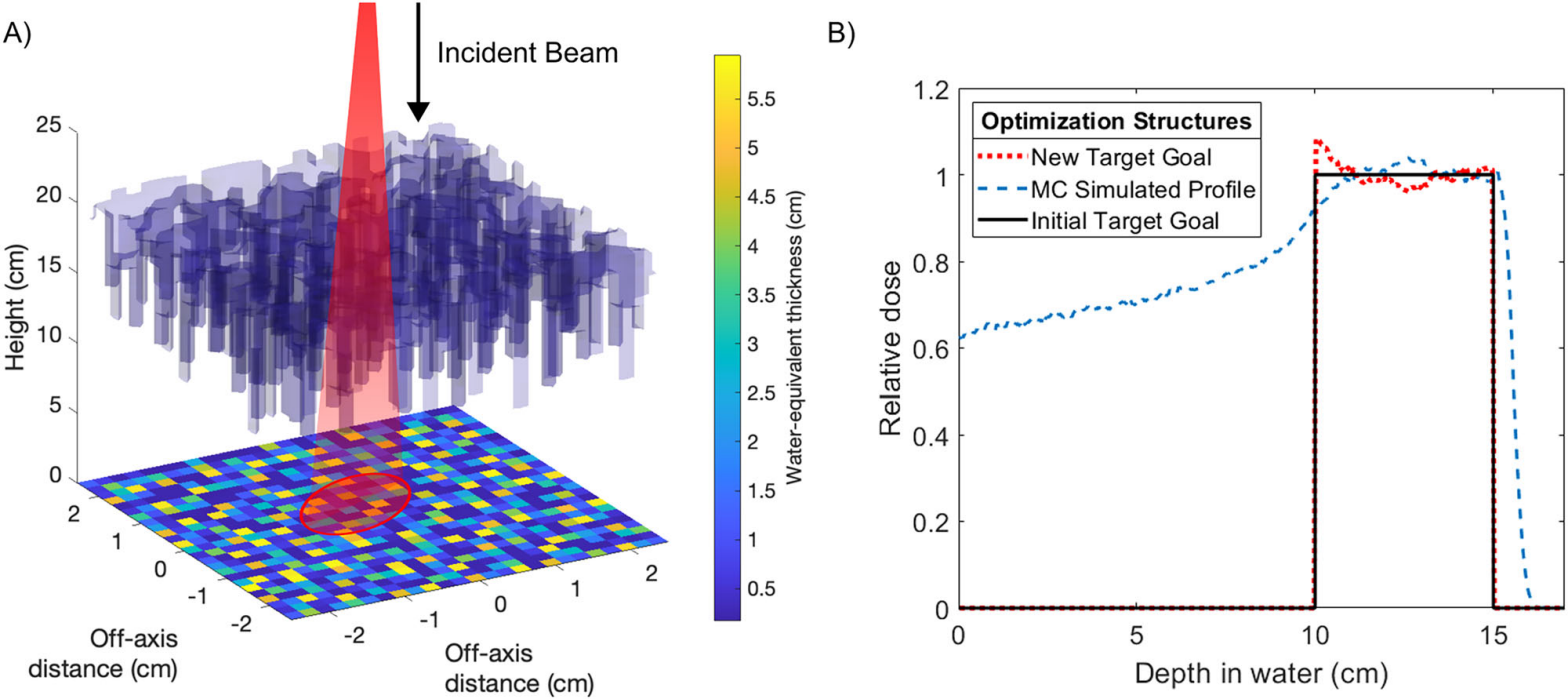
Depiction of the pillar height optimization technique used to create uniform spread-out Bragg peak profiles. The effective depth dose distribution within a region of the SpeleoFilter is dependent on the distribution of surrounding pillar heights and the residual range loss incurred from the protons that traverse through this region, shown as a projected beam that intersects a collection of pillars whose water-equivalent thicknesses (**A**). The resultant depth dose distribution within a region of the SpeleoFilter is estimated as the superposition of the individual depth dose profiles caused by each pillar and the nominal beam energy (**B**). This process seeks to match a desired SOBP width and depth, shown as the initial goal profile (black solid line). However, this process neglects alterations in scattering conditions which are accounted for by augmenting the depth dose goal (red dotted line) to reflect these conditions within the SpeleoFilter that are simulated using Monte Carlo methods (blue dashed line).

Simple phantom geometries consisting of a uniform cubic and cylindrical water phantom geometries were initially used to evaluate the dosimetric characteristics of fixed SpeleoFilters to generate the necessary energy modulation of sufficient resolution to deliver clinically relevant and useful dose distributions. As illustrated in Fig. 2, SpeleoFilters were first optimized to provide SOBP profiles ranging between 3 cm and 7 cm in length for a uniformly scanned proton beam delivered over a 5 cm × 5 cm field. The distribution of pillars and their heights were optimized specific to these goals using scripts written in MATLAB^23^, which calculated resulting dose distributions using a set of executables to run the Monte Carlo simulation package developed for this work. Individual pillars were modeled as a contiguous rectangular column with a 1 mm square base dimensions extruding from a 1 mm thick base layer. To a first-order approximation, it was assumed for this exercise that the depth dose distribution produced within a region of the SpeleoFitler could be determined from the superposition of depth dose profiles from a collection of individual pillars within a specified areal region projected through the SpeleoFilter. The corresponding depth dose distributions produced within this region of the SpeleoFitler were calculated by resampling the nominal depth dose distribution of the nominal proton beam energy by the amount of water-equivalent material a proton traverses through each pillar. Each iteration of the optimization computes the squared differences between the dose distribution using a SpeleoFilter to all points within the targeted dose profile. If the objective function improves, then the change to the SpeleoFilter’s pillar heights are accepted and otherwise rejected. The process continues until all pillars within the SpeleoFilter have been evaluated. Afterwards, the changes are compiled into an updated SpeleoFilter that becomes the new reference, and the process repeats for a new iteration until the specified number of iterations are completed.

#### Fixed width SpeleoFilter proton arc phantom planning study

A full and partial PBS proton arc treatment delivery were simulated using a fixed SpeleoFilter that was optimized to produce a 5 cm SOBP in water, independent of any patient-specific variation. These SpeleoFilter treatment plans were generated using a 150 MeV proton beam in combination with a SpeleoFilter that produces a nominal 5 cm SOBP to cover a cylindrical target centered within a 25 cm diameter cylindrical water phantom along the central axis. Each treatment was optimized from a set of beamlets delivered across the entire surface of the SpeleoFilter with 2.5 mm spot spacing contiguously in 1*°* increments, permitting the optimizer to utilize any part of the SpeleoFilter at any gantry angle to optimize the dose distribution. Plans were also created for a 5 cm off-axis target, simulating a more complex scenario where pathlength differences between the phantom surface and the target varied across the arc. For this exercise, the same 5 cm SOBP SpeleoFilter was used with a minimal number of energy changes to compensate for the path length differences, including a 119.1 MeV, 128.9 Mev, and 136.9 MeV proton beam energy that were partitioned among angular segments between 0*°* and 53*°*, 53*°* and 74*°*, and 74 and 90*°*, respectively. For comparison, a set of corresponding IMPT treatment plans were created as a baseline comparison which used an equivalent scanning field size, lateral spot spacing, and 2.5 MeV energy spacing. Beam energies for the IMPT plans ranged between 100 MeV and 150 MeV for the centrally located target and 77 MeV and 141.5 MeV for the offset target. An alternative method of optimizing the SpeleoFilter for pathlength differences is later presented using a patient-specific example.

Individual beamlets were simulated with 10E5 histories using the Geant4 simulation executable to generate a library of beamlet profiles with a $1$ mm × 1mm lateral and 5 mm longitudinal dose grid resolution whose intensities were optimized using a least-squares minimization approach for IMPT^24^. Four regions were considered for the purposes of this optimization. The 5 cm diameter target was optimized so that 95% of its volume received 50 Gy. The skin organ was modeled as the inner 5 mm region from the outer perimeter. High- and low-dose conformity, defined as the ratio of the 90% and 50% isodose lines, respectively, to the target volume that was completely encapsulated by respective isodose lines were quantified from a 10 mm rind of healthy tissue immediately adjacent to the intended target.

#### Patient-specific SpeleoFilter treatment planning study

**Fig. 3:**
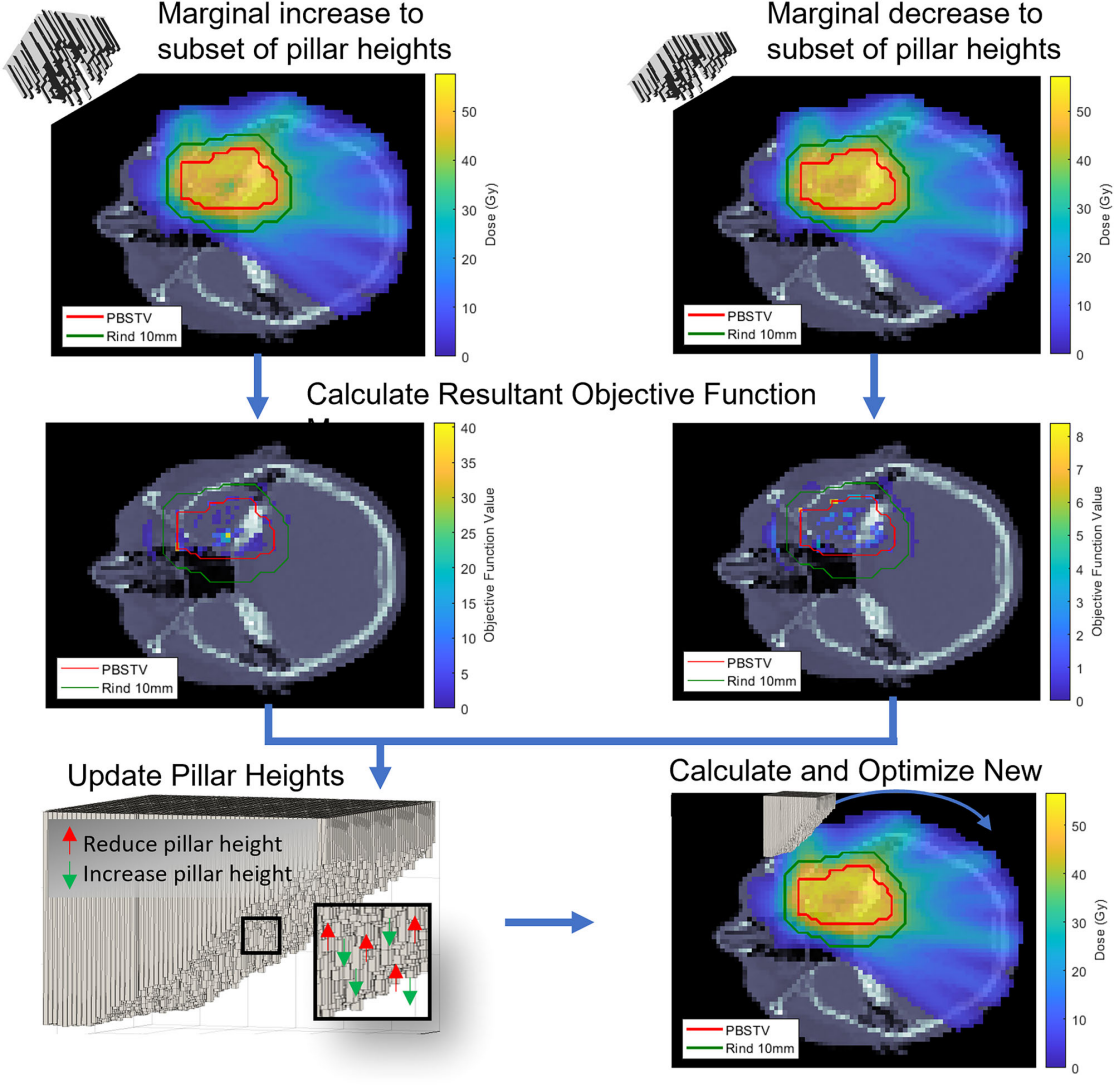
Illustration of the optimization process used to optimize the SpeleoFilter Pillar heights and beam intensity modulation for a proton arc delivery. Each iteration the dose distribution is simulated where a portion of the SpeleoFilter pillar heights is increased (highlighted in red) or decreased (highlighted in green) relative to the initial SpeleoFilter design. Spatial changes to the dose distribution are quantified based on the calculated objective function used to optimize the beamlet intensity modulation. Changes to the objective function are calculated for each incremental change in pillar height for the entire dose distribution, which are used to determine the new SpeleoFilter pillar heights.

Direct, patient-specific SpeleoFilter optimization was performed for an anonymized CT planning dataset supplied as part of a mutual confidential disclosure agreement between the University of Iowa and University of Pennsylvania, which had obtained this data through an existing institutional review board (IRB) approved study where informed consent was obtained. It was the intent of this exercise to develop the technical infrastructure to demonstrate, via proof-of-concept, how a single SpeleoFilter could be optimized uniquely to the patient for a proton arc treatment and maintain comparable dosimetric quality to that of a standard IMPT technique. Figure 3 illustrates the planning and optimization methods used for this exercise. Unlike the methods discussed in “Methods and materials,” section, the beamlet intensity modulation and SpeleoFilter pillar distribution were optimized cohesively using an in-house treatment planning system based on a least-squares optimizer combined with the Monte Carlo simulation framework developed in the work.

The proton arc delivery was initialized using a 160 MeV beam energy and a 120*°* arc extending occipitally from the patient’s right lateral side. Spot positions were placed across the range of the arc to cover the lateral extent of the target at any beam angle within the beam’s-eye-view (BEV). A primary set of spots were placed using a 5 mm spot spacing in the BEV. It was assumed that the rectangular grid was raster scanned perpendicular to the direction of the rotational arc, where each column of spots is scanned at a discrete angle, roughly 10*°* for every 5 mm in the BEV for the case presented. At each discrete angle a secondary set of spot positions are placed 2.5 mm and 5 mm laterally so that a 10 mm region, rather than a single row, is scanned at each discrete angle. The SpeleoFilter was initialized with pillar heights that centered the beam range within the target across the arc.

During each stage of the optimization, a library of beamlet dose profiles were simulated for three different scenarios including the current iteration of the SpeleoFilter, a systematic increase to a randomized subset of pillar heights, and a systematic decrease to the same subset of pillar heights. Calculations were performed using the Monte Carlo framework that include a complete model of the treatment delivery with the IBA DN model. This included the patient geometry reconstructed from the CT dataset and a model of the SpeleoFilter that was positioned in place of an external range shifter within the DCS. Beamlet intensities from the nominal SpeleoFilter design were optimized to achieve the dosimetric goals to the target and regions of interest using a least-squares minimization optimization^24^, which were then applied to the simulated treatment deliveries of the modified pillar heights. An objective function map was calculated for each of the three simulated scenarios reflecting the deviation to the planning goals of the treatment. Improvements to the objective function map were evaluated for each candidate change in the pillars’ heights, which were updated using the gradient of this map to determine the new pillar heights for the next iteration. This process is repeated until the objective function could no longer be reduced.

The resultant dose distribution delivered from the optimized SpeleoFilter was evaluated for plan quality and sensitivity to production errors. A treatment planning comparison was performed by simulating a two-field IMPT treatment plan with the same patient and beamline geometry, lateral and distal spot spacing. Treatment plans were evaluated for their differences in target coverage and overall conformity using concentric rings. A worst-case scenario approach was used to evaluate the sensitivity of the delivered dose distribution due to production errors of a SpeleoFilter. Simulations were repeated using SpeleoFilters whose pillar heights were systematically increased or decreased by 0.5 mm and 1 mm, which were considered conservative values far exceeding the reported 3D printing tolerance of 0.127 mm as reported by the manufacturer, Stratasys.

### Reporting summary

Further information on research design is available in the Nature Portfolio Reporting Summary linked to this article.

## Results

### Experimental validation

**Fig. 4:**
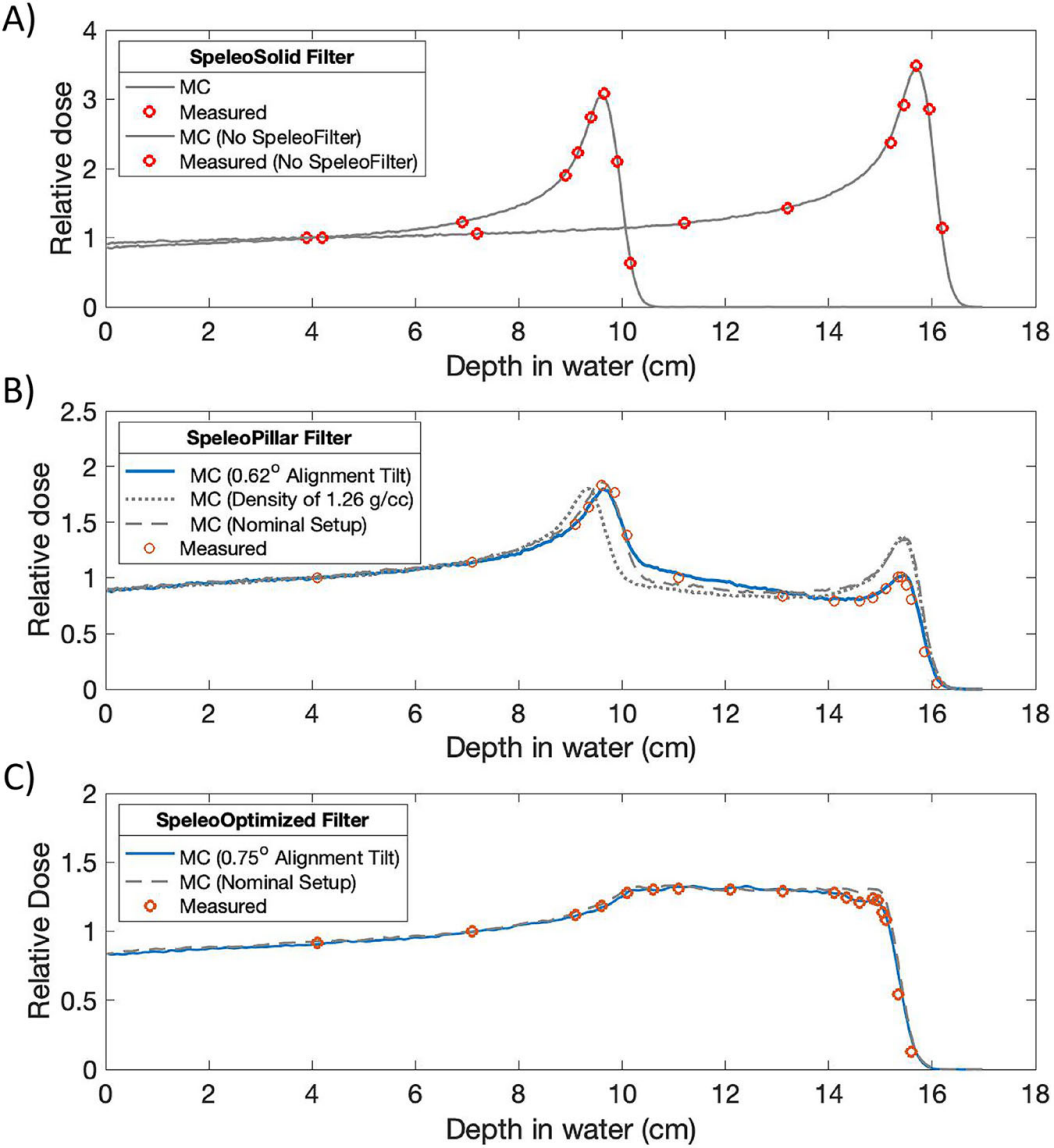
Comparison of measured and simulated depth dose distributions from a set of experimental SpeleoFilter prototypes. Measurements performed using an IBA PPC05 ionization chamber and Geant4-simulated depth dose distribution are shown for a solid SpeleoFilter (SpeleoSolid, **A**), a SpeleoFilter consisting of pillars of alternating heights (SpeleoPillar, **B**), and an optimized SpeleoFilter to provide a nominal 5 cm uniform dose profile (SpeleoOptimized, **C**). Additional profiles were simulated to show the sensitivity of each profile to the assumed material density and setup error. Unless otherwise noted, a nominal material density of 1.19 g cm^−3^ and initial alignment of the SpeleoFilter normal to the axis of the delivered field were assumed.

Dose measurements were acquired using the IBA DN system at the Miami Cancer Institute along the central axis at specified depths within a water phantom for each prototype SpeleoFilter. Figure 4 shows the measured and simulated dose profiles used to validate the Monte Carlo model for the SpeleoSolid, SpeleoPillar, and the SpeleoOptimized SpeleoFilters. Great agreement was observed between the computerized model and scanned surface of the 3D printed SpeleoOptimized filter with a DICE similarity coefficient of 0.978 with 95% of the surface having a Hausdorff distance of less than 0.5 mm. The CT-measured density from the SpeleoSolid was within 0.84% of the manufacturer-reported density. The pristine Bragg peak profile that was measured for an unfiltered 150 MeV beam showed excellent agreement with the simulated profile with all measurement points matching the Monte Carlo simulated SpeleoSolid profile to within a 1%/1 mm gamma criteria. Additional Monte Carlo simulations were run by changing the nominal density and orientation of the SpeleoSolid filter to investigate the impact on the resultant depth dose profile. As shown in Fig. 4, changes to the material density caused the relative separation of the peaks to change while any deflection of the SpeleoFilter normal to the direction of the scanned beam caused changes to the relative intensity of the two peaks. A nominal density of 1.19 g cm^-3^ with a SpeleoFilter setup error of 0.62*°* rotated about the X- and Y- plane resulted in a Monte-Carlo generated profile that matched all measurement points to within a 2%/1 mm gamma criteria. The measured and Monte Carlo simulated profiles for the SpeleoOptimized SpeleoFilter are plotted in Fig. 4 assuming the nominal material density and 0.75*°* angled off set. All measurement points matched with the Monte Carlo simulated profile to within a 1%/1 mm gamma criteria and achieved a uniformity of 3% of the mean across the dose profile.

### Optimized Uniformity

**Fig. 5:**
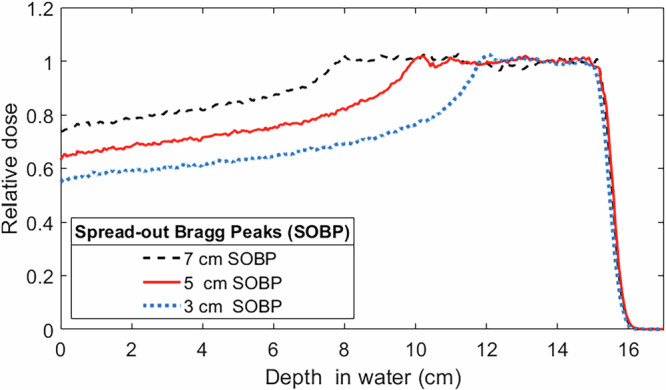
Spread-out Bragg peak (SOBP) profiles from a SpeleoFilter. Uniform depth dose profiles optimized to provide a SOBP range of 3 cm, 5 cm, and 7 cm along the central axis. Each profile was simulated in Geant4 for a uniform field delivered using pencil beam scanning.

A variety of Bragg peak profiles can be optimized for a given SpeleoFilter. To demonstrate proof-of-concept, a series of SpeleoFilters were optimized to provide a 3 cm, 5 cm, and 7 cm uniform spread-out Bragg peak profile for a nominal 150 MeV proton beam and a lateral beam-scanned area of 5 cm $\times$ 5 cm. Figure 4 shows the resultant profile for each SpeleoFilter design, which were evaluated from an independent Monte Carlo simulation utilizing enough simulation histories to achieve less than a 1% simulation error within the central uniform dose region. The distribution of pillar heights used to achieve each depth dose profile are plotted in Fig. 5. A maximum deviation within the dose profile uniformity was 2.45%, 2.53% and 3.56% from the mean for the 3 cm, 5 cm, and 7 cm uniform dose profile SpeleoFilter design, respectively, with over 95% of the dose profile maintaining a uniformity of 2.5% for each SpeleoFilter.

### Fixed width SpeleoFilter proton arc phantom planning study

**Fig. 6:**
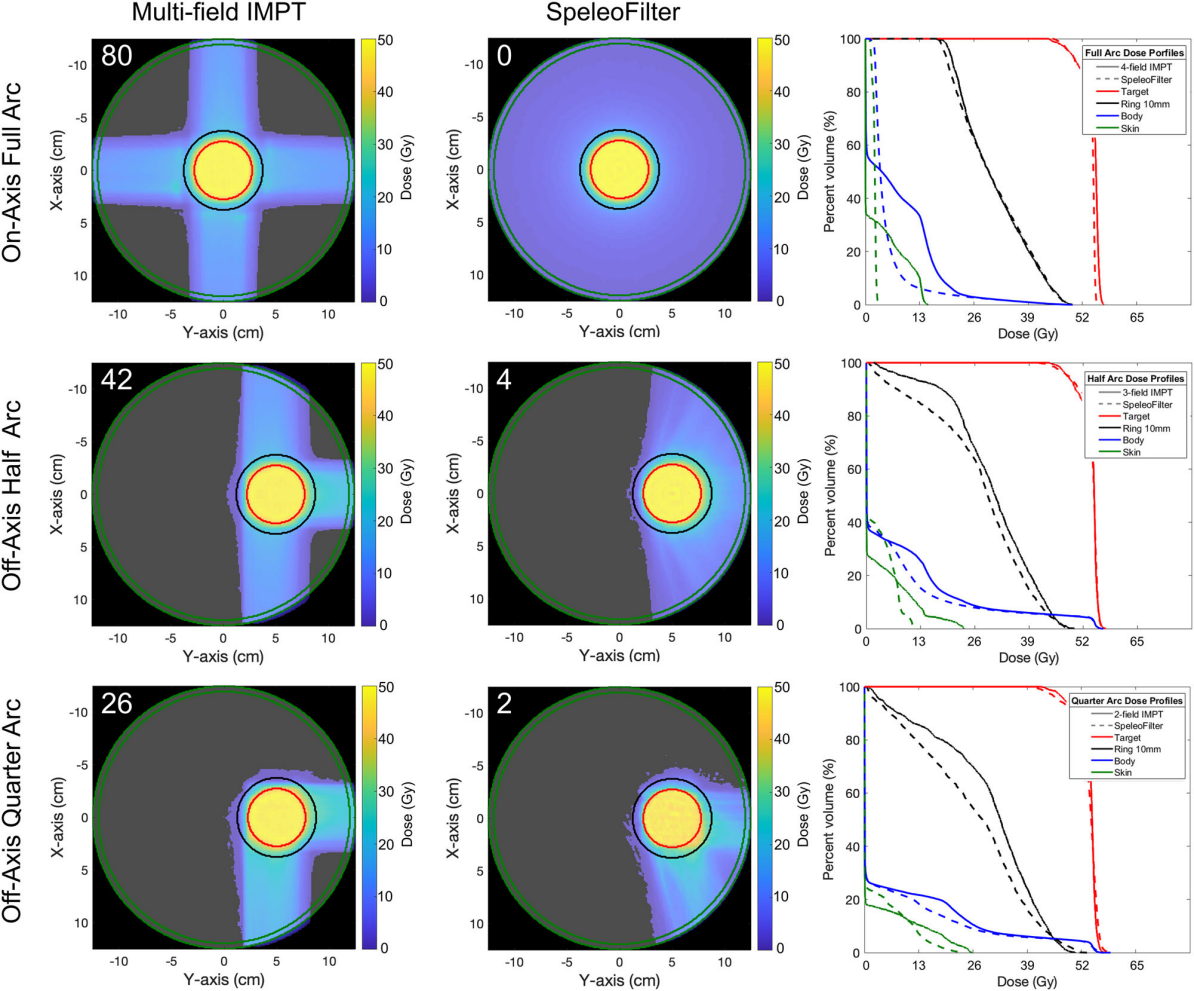
Dose profiles of the full and partial arc treatments delivered with a 5 cm SpeleoFilter (center row) compared against multifield IMPT treatments (left row). The number of energy changes are listed in the top corner for each delivery. Dose volume histograms are shown for the four-field IMPT against the full-arc (top row), three-field IMPT against a half-arc (center row), and the two-field IMPT against a quarter arc (bottom row). Regions of interest are shown for the skin (green), pencil beam scanning target volume (Target, red), the adjacent 10 mm of surrounding normal tissue (Ring 10 mm, black), and the body volume (blue).

Multifield IMPT and SpeleoFilter arc treatment plans were generated using the Monte Carlo methods for a cylindrical phantom. Off-axis target treatments were planned using the three-field (180*°* span angle) or two-field (90*°* span angle) IMPT technique that was matched against a half- or quarter-arc treatment, respectively, using the SpeleoFilter. A four-field IMPT and full-arc SpeleoFilter treatment plan were compared for a centrally located target. Dose profiles for each plan are shown in Fig. 6. Plans optimized using a SpeleoFilter resulted in comparable or superior treatment planning quality to IMPT as summarized in Table 1.

**Table 1:** Dose distribution summary, normalized to 50 Gy coverage, of each delivery shown in Fig. 5

Delivery	Energy	Conformity Index		Max Skin	Integral Dose
Method	Changes	90%	50%	Dose (Gy)	(10^-3^ Gym^3^)
4-Field IMPT	80	1.059	1.617	15.23	1.800
3-Field IMPT	42	1.070	1.930	24.27	1.255
2-Field IMPT	26	1.088	2.487	26.85	1.121
Full Arc	0	1.068	1.555	2.78	1.342
Half Arc	4	1.036	1.643	12.62	0.972
Quarter Arc	2	1.082	2.063	24.86	0.929

Depending on the field arrangement and arc span, the high dose conformity remained similar among IMPT and arc treatments delivered using a SpeleoFilter ranging between 1.059-1.088 and 1.036-1.06, respectively, with smaller span angles and fewer arcs resulting in a reduced high-dose conformity. Low-dose conformity was improved by 17%, 15% and 4% for the quarter-, half-, and full-arc deliveries, respectively, using a rotational arc and a SpeleoFilter. Similarly, the maximum skin dose and integral body dose were all improved using the rotational arc delivery and SpeleoFilter over a multi-field IMPT techniques. The maximum skin dose sparing ranged between 81%, 48%, and 7% for the full, half, and partial arcs while the integral dose sparing was improved by 25%, 23% and 17% compared to the four, three, and two-field IMPT treatment respectively.

### Patient-specific SpeleoFilter treatment planning study

**Fig. 7:**
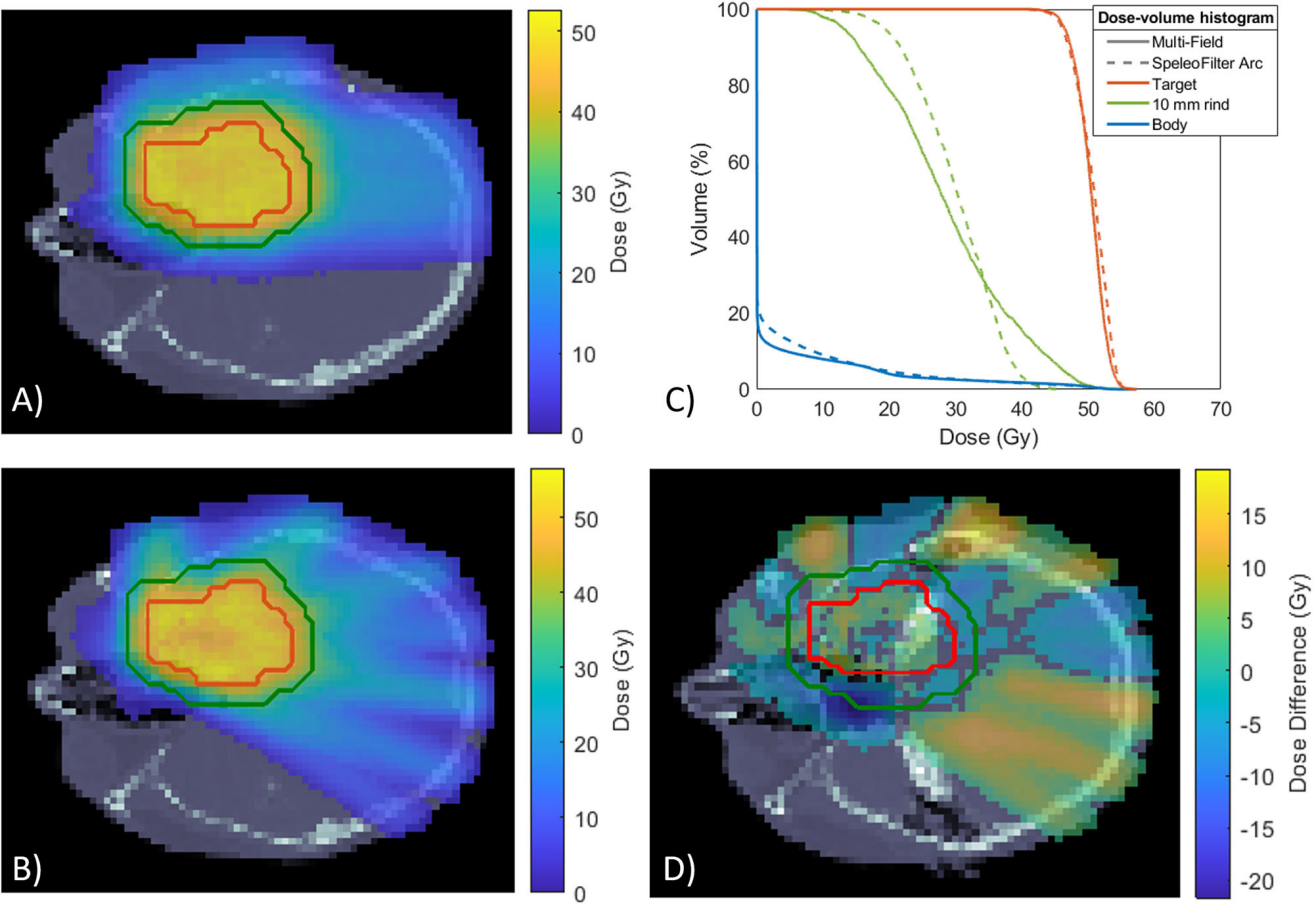
The dose distributions delivered with proton arc and a SpeleoFilter can achieve similar plan qualities compared to intensity modulated proton therapy (IMPT). Monte Carlo simulated dose profiles are shown for a two-field IMPT (**A**), a proton arc delivery using a SpeleoFilter (**B**), the dose-volume histogram (DVH) (**C**), and the difference profile between these two delivery techniques (**D**). The legend provided for the DVH lists the dose coverage plots for the pencil beam scanning target volume (PBSTV, red), body (blue), and surrounding 10 mm rind of nearby healthy tissue (green).

**Fig. 8:**
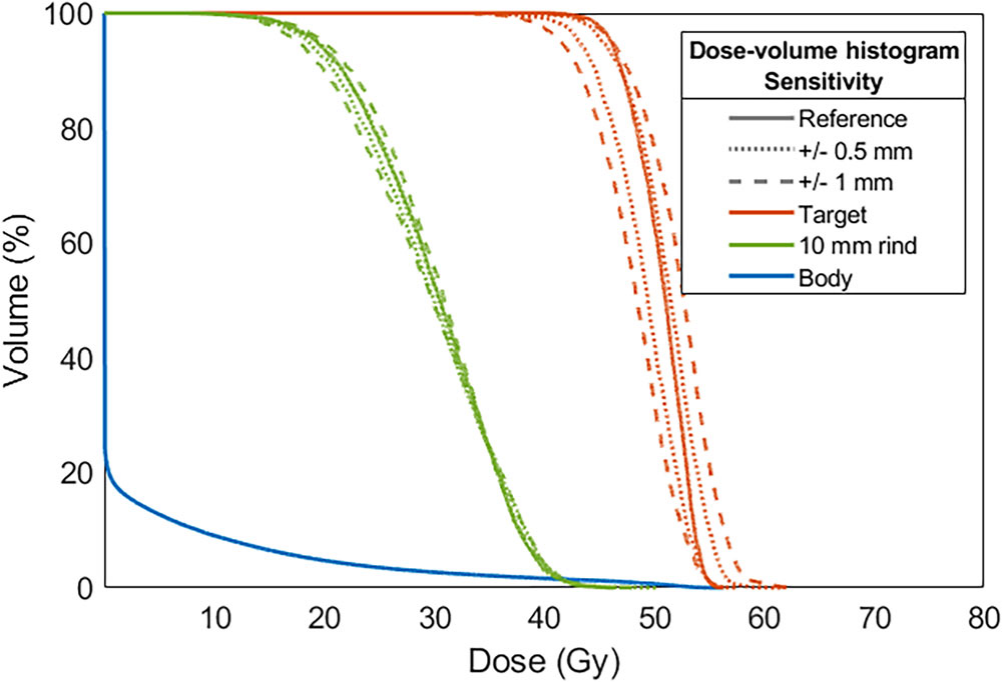
Error-sensitivity analysis results illustrating the potential variability of target coverage and organ doses due to systematic errors in SpeleoFilter construction. SpeleoFilters exhibit a moderate sensitivity to construction errors. Changes to the pencil beam scanning target volume (PBSTV, red) and the surrounding 10 mm rind of healthy tissue (green) were simulated for systematic changes to the pillar heights of 0.5 mm (dotted line) and 1.0 mm (dashed lines) across the entire SpeleoFilter. Decreasing pillar height resulted in an increased target maximum dose while increasing pillar height resulted in reduced target coverage.

Proton arc treatments aided in the delivery using a SpeleoFilter can provide dosimetrically comparable treatment quality compared to multi-field IMPT. Figure 7 illustrates the comparison of a fixed, two-field IMPT and SpeleoFilter PBS proton arc treatment planned using Monte Carlo calculation methods on a clinical dataset. Dosimetrically, both treatments achieved similar target coverage, exhibiting a characteristic trend of arc-style deliveries resulting in a trade-off between increased low-dose volume in favor of improved high-dose conformity. The IMPT treatment utilized a total of 3793 spots and 45 energy layers whereas the SpeleoFilter arc delivered a more efficient treatment that utilized 612 spot positions and a single beam energy. Furthermore, both treatments required roughly the same output to deliver based in the Monte Carlo simulated dose profiles. The ratio quantifying the relative the sum of weights to deliver the SpeleoFilter proton arc to the IMPT treatment plan, which is equal to the total number of protons delivered, was 0.9858. Proton arc treatments delivered using a SpeleoFilter were sensitive to errors in pillar heights. The DVH impact of systematic errors to the 3D printed SpeleoFilter are shown for up to 1 mm in Fig. 8. Positive errors in pillar heights showed a greater sensitivity than negative errors. Systematically increasing all pillar heights across the SpeleoFilter reduced the beam range across the arc resulting in a 6% reduction to the volume of the target receiving prescription coverage with no statistically significant change to the maximum dose to the target. Systematically reducing the pillar heights by 0.5 mm across the SpeleoFilter increased the maximum dose received by 1 cc of the target by 3% with statistically no significant change in the prescription coverage of the target. Systematic changes to the reported 3D printing tolerances reported by Stratasys had no statistically significant effect on the resulting DVH.

## Discussion

The pillar-like features of a SpeleoFilter enable efficient proton arc therapy through the delivery of a polyenergetic spot scanned proton beam, thus minimizing the number of energy changes and redundant beam scanning throughout the arc. Another unique feature of a SpeleoFilter is the inclusion of local variability among pillar heights that provides the necessary depth dose distribution across the arc to achieve the desired dosimetric planning goals while preventing patterns of locally hot and cold streaks from occurring that are common with ridge filters^11^. In contrast to some designs that utilize Ziggurat-shaped pins^13^, Speleofilters spatially discretize an optimized beam energy distribution across the treatment field by producing numerous sub-millimeter minibeamlets from a single scanned beam. The pillars protruding from the base are also spatially distinct enabling a spatially variant depth-dose profile across the SpeleoFilter without the need for a tertiary compensator. Unlike fixed ridge filters, the shape of the SpeleoFilter and its resulting dose distribution can be optimized cohesively with the PBS spot map directly from dosimetric goals and the patient’s anatomy.

Two applications of SpeleoFilters are presented in the work. Initially an approach is presented in Fig. 6 for a simplified phantom geometry where similar, if not improved, plan quality was achieved using a rotational arc delivery and a SpeleoFilter compared to IMPT. This example intentionally utilized a SpeleoFilter with a uniform depth dose profile resulting in a 5 cm SOBP. For the case presented, it was possible to achieve adequate target coverage under different delivery scenarios using a common SpeleoFilter with minimal energy transitions. This method may also have logistical advantageous, as the SpeleoFilters would not require additional quality control for each patient as they do not change. Clinically, this may be translated to a system consisting of a common set of interchangeable SpeleoFilters where treatments are planned by optimizing the sequencing of Filters during treatment to minimize energy transitions. Alternatively, this technology is well suited for patient-specific treatments where one or more SpeleoFilters are optimized uniquely to the patient to provide the best plan quality and delivery efficiency. This is demonstrated for a patient-specific device in Fig. 7 that uses a fraction of the delivered beam spots with a single beam energy compared to its IMPT counterpart, effectively resulting in a treatment duration that is limited by the rotational speed of the gantry.

SpeleoFilters are sensitive to 3D printing and alignment errors, requiring both accurate and precise 3D printing methods to match the planned distribution of pillar heights and ensure their alignment with the beam during treatment. Deviations from the planned SpeleoFilter design or beam alignment can affect the spatial distribution of energy modulation, which in turn affects the delivered dose distribution in a similar manner to how range uncertainties can impact the dose distribution in PBS proton therapy. However, as it appears in this work, sufficient 3D printing accuracy is possible to satisfy clinical standards. Small changes to the SpeleoFilter’s normal alignment to the beam’s central axis can also impact the delivered dose distribution as shown in Fig. 4 where manual alignment errors a 0.75° alignment offset resulted in a 2% dose deviation towards the distal portion of the measured PDD. Future improvements to the mechanical range shifter mount within the DCS will likely provide the necessary means to ensure accurate and reproducible alignment based on the strict machining tolerances of the DCS. This has already demonstrated this for the general mounting and isocentric alignment accuracy for the DCS within a clinical PBS beamline, which has shown sub-millimeter mounting reproducibility and sub-degree collimator focusing accuracy^25–27^. Robust optimization methods could also be implemented into the optimization process to minimize the impact of 3D printing and alignment errors on the delivered dose distribution. A potential strategy based on the sensitivity results in Fig. 8 would be to penalize large changes among adjacent pillars and bias under printing, rather than over printing, of pillar heights as it appears to have a lesser impact on the overall dose distribution. In addition to their generalizability among a multitude of treatment options, the 3D-printing process utilizes materials that are expected to have other clinical benefits such as low neutron production and a low footprint allowing for a compact design that can be integrated with state-of-the-art PBS collimators, preserving a powerful upcoming feature in modern PBS^28–32^.

It should be emphasized that the results from this work demonstrate feasibility. As such, most of the results presented in this work are confined to phantom geometries to allow for experimental validation of the Monte Carlo framework. Future work will be aimed to demonstrate broader clinical translation and quantify utility among multiple treatment sites. While great agreement was observed between the Monte Carlo methods and experimental profiles, more rigorous quality assurance protocols will need to be developed for routine clinical use, such as quality control methods to independently verify 3D printing accuracy and quality assurance methods to verify deliverability prior to treatment. Furthermore, this work presents a limited embodiment of how the mathematical optimization framework could be implemented. Thus, these methods show only proof-of-concept and may not currently reflect the optimal sequencing of how the SpeleoFilter shape and beamlet weight optimization could be performed, especially for more complex geometries inherent to human anatomy or the use of multiple SpeleoFilters portioned across an arc to further improve plan quality. The additional Monte Carlo study presented in Fig. 7 was performed using a patient dataset to help bridge this disparity. Future work will continue to consolidate the treatment planning process to include energy-specific collimation, robust optimization to account for 3D printing and delivery errors, as well as to evaluate the capacity of these methods for a larger cohort of patient treatment plans. The Monte Carlo methods developed for this work are an important foundation, allowing for future development of planning strategies that will benefit from calculations that accurately account for the inherent heterogeneities within the SpeleoFilter and patient geometry. Such an integrated framework is essential to enable future clinical treatment planning investigations using SpeleoFilters for proton arc deliveries and integrating this technology with the current state-of-the-art systems.

## Supplementary information

Reporting Summary

## Data Availability

Portions of the data used in this study are available upon reasonable request to the corresponding author. Clinical information used in this study cannot be shared outside of the institution-approved IRB disclosure agreement.

## Code availability

The algorithms used in this study are currently under patent pending status and specific code is not publicly available at this time. Licensed access may be arranged upon reasonable request to the corresponding author.
